# Group discussions on how to implement a participatory process in climate adaptation planning: a case study in Malaysia

**DOI:** 10.1016/j.ecolecon.2020.106791

**Published:** 2020-11

**Authors:** Valentina Palermo, Yeray Hernandez

**Affiliations:** European Commission, Joint Research Centre, Via Enrico Fermi 2749, I, 21027 Ispra (VA), Italy

**Keywords:** Climate change, Adaptation, Stakeholder and citizen participation, Focus groups, Global Covenant of Mayors (GCoM)

## Abstract

The frequency and intensity of extreme climate events are increasing all around the world, due to climate change. Climate adaptation strategies are therefore needed, since mitigation strategies alone are not sufficient to avoid serious impacts of climate change. However, adaptation to climate change is not straightforward, as it is highly influenced by diverse and conflicting interests as well as epistemological (or scientific) uncertainties. Therefore, a minimum requirement for its success is the active participation of stakeholders and citizens in the adaptation policy cycle. This paper presents a case study on a participatory process involving civil servants from different municipalities in Malaysia, in Southeast Asia, with a view to considering the optimal level of engagement that is required for climate adaptation planning. The exercise consisted of a Focus Group session, where participants were asked to discuss the level of stakeholder and citizen participation that should be adopted within the Global Covenant of Mayors for Climate and Energy initiative. Contrary to authors' expectations, the participants tended to suggest medium to high levels of participation in the planning process. During the dialogues, a walking activity through the city, aimed at identifying hotspots of climate risks and defined as “safety walks”, was one of the ideas proposed as a high-potential participatory method, spreading in the adaptation framework. Safety walks could complement climate modelling and enhance the robustness of climate risk assessments.

## Highlights

• A focus group session on stakeholders participation in adaptation planning
• Medium-high levels of participation in the planning process were suggested.
• Technical expertise from local community is needed
• "Safety walks" suggested as a high-potential stakeholder-involving participatory technique
• Site-specific challenging issues were addressed through active discussion and best practices

## 1 Introduction

Climate change is triggering more frequent and intense extreme climate events all around the world. Warm days and nights have increased at the global scale, heatwaves have become more common in large parts of Europe, Asia and Australia, and heavy precipitation events have increased on average, implying greater risk of flooding. Droughts, cyclones and wildfires are threatening the most vulnerable human systems (IPCC, 2014). These climate-related risks and potential impacts are foreseen to grow in the future, especially affecting disadvantaged people. Extreme weather events will undermine food security, impacting people's lives, and weakening economic activities, not to mention environmental impacts, such as modified hydrological systems or changes in species abundance. A more in-depth analysis of climate risks and impacts in the Malaysian context is provided in Section 2 below.

Human activities have already warmed the planet by around 1°C above pre-industrial levels, while global warming is expected to reach 1.5°C–2°C somewhere between 2030 and 2050, implying even more frequent and intense climate events and impacts than today (IPCC, 2018). The magnitude of the climate risks and possible impacts will depend on the extent of climate action, which can be promoted also by non-State actors and local governments, as was highlighted at the 2015 United Nations Climate Change Conference, or COP 21, in Paris (Bertoldi, 2018; Melica et al., 2018). However, even though decision-makers at COP 21 agreed to keep a global temperature rise this century well below 2 °C (above pre-industrial levels) and to pursue efforts to limit the temperature increase even further to 1.5°C,^2^ the nationally determined contributions (NDCs) which are at the heart of the Paris Agreement are not sufficient to meet the committed goals of the Agreement, leading to a 3–4°C world (du Pont and Meinshausen, 2018; Nieto et al., 2018). Consequently, there is the risk of ending up in “Hothouse Earth”, as described by Schröder and Storm (2018).

As defined by Field et al. (2014), climate adaptation refers to the process of adjustment to actual or expected climate and its effects. According to the authors: “In human systems, adaptation seeks to moderate or avoid harm or exploit beneficial opportunities. In some natural systems, human intervention may facilitate adjustment to expected climate and its effects”. Faced with the climate challenge, adaptation strategies are needed, since mitigation strategies alone are not sufficient to avoid serious climate change impacts (Biesbroek et al., 2009; Swart and Raes, 2007). The integrated mitigation and adaptation approach enables a multi-level governance with many and various spatial approaches in addressing climate impacts (Biesbroek et al., 2009; Gupta, 2007; Klein et al., 2005). Adaptation is expected to be more challenging for ecosystems and for food and health systems, at warmer scenarios beyond 1.5°C (IPCC, 2018). Examples of adaptation strategies include early warning systems for heat, mangrove restoration to reduce flood risks and protect shorelines from storm surges, elevation of concrete floors against extreme rainfall, building low aerodynamic houses with palm leaves as roofing against cyclones, beach nourishment and grey infrastructure as flood defence, and insurance systems to protect farmers from yield losses (Field et al., 2014).

Climate adaptation is highly influenced by diverse and conflicting interests, as well as by “epistemological” (or scientific) uncertainties which are produced - as stated by Funtowicz and Ravetz (1993) - when irremediable uncertainty is at the core of the problem, as when computer modellers recognize ‘completeness uncertainties’ which can vitiate [i.e. impair] the whole exercise. With regards to conflicting interests, it is known that the design of climate adaptation policies is shaped by how people and organizations observe risks and uncertainties (IPCC, 2014). For example, different stakeholders tend to perceive the impacts of extreme weather events differently. While some stakeholders only see direct damages to infrastructure, others identify impacts on businesses or quality of life (Becker et al., 2015). Community values and beliefs are also important to climate adaptation: for instance, actual experience of climate-related hazards usually motivates people to support adaptation (Brink and Wamsler, 2019). Gender matters too, since males tend to be more interested in economic values than females, the latter tending to be more interested in social values (Brink and Wamsler, 2019). Moreover, citizens are usually more aligned and satisfied with environmental organizations and scientists than local authorities and central governments, indicating possible conflicts (Zerva et al., 2018). Sometimes, climate change professionals have diverse points of view on how adaptation should be implemented (Nelson et al., 2016).

On the other hand, uncertainties are mainly related to limitations on how to measure rare events, as well as the challenges of evaluating causality in complex or multi-component processes that can span physical, biological and human systems (IPCC, 2014). Consequently, climate change, its effects, and the possible actions fall into the field of “post-normal science”, defined as a problem-solving strategy to manage environmental risks that imply high system uncertainties and high decision stakes (Funtowicz and Ravetz, 1993). Uncertainties are either epistemological (see above) or ethical (for example, the debate on climate migration has many social and political implications), whereas the stakes reflect potential conflicts among different stakeholders, as indicated. Funtowicz and Ravetz (1993) consider that post-normal science problems, such as climate adaptation, can be addressed by including participants in the process, which can ensure high quality scientific outputs. Furthermore, the direct engagement of legitimated and skilled people broadens and enriches the process since - as stated by Funtowicz and Ravetz (1993) - persons directly affected by an environmental problem will have a keener awareness of its symptoms, and a more pressing concern with the quality of official reassurances, than those in any other role.

The need for public participation in climate adaptation planning is recognized as fundamental by the specialized literature on the topic. For example, adaptation governance that involves citizens enhances sustainable climate adaptation (Wamsler and Raggers, 2018) and capacity (Sarzynski, 2015). Citizen engagement may even reframe fundamental research questions and make them more meaningful (Hernandez et al., 2018). It has also been recognised that citizens and stakeholders have something to say regarding resources and environmental modelling (Voinov et al., 2016). Another example is citizen science, which is known to contribute effectively to climate adaptation governance by means of decision-making support (Bremer et al., 2019). However, it has also been said that citizen engagement may hinder sustainable action (Wamsler et al., 2019), possibly as a consequence of distrust, delay and entrenchened positions (Saengsupavanich et al., 2012). Section 3.1.3 gives details on downsides of citizen engagement.

This paper presents a case study on a participatory process based on a Focus Group session, as a means to reflect on the ideal level of citizen and stakeholder engagement in climate adaptation planning. The exercise was carried out during a training session held in Johor Bahru (Malaysia), and was aimed at civil servants within the framework of the Global Covenant of Mayors for Climate and Energy initiative (GCoM). Our research hypothesis was that civil servants (as stakeholders of the Public Administration) would tend to suggest lower levels of stakeholder and citizen participation in the planning process. The results were surprising and potentially useful for the climate adaptation community. Although other researchers have conducted participatory exercises with climate practitioners to reflect on participation in climate adaptation planning (Göpfert et al., 2019; Wamsler et al., 2019), to the authors' knowledge this was the first time that a participatory exercise was carried out to reflect on the suitable level of stakeholder and citizen participation in the climate adaptation planning cycle.

The remainder of the paper is organised as follows: the context for the case study is introduced in Section 2; the approaches that were applied are described in Section 3; the results are presented in Section 4 and discussed in Section 5; the conclusions of the case study are presented in Section 6.

## 2 Malaysia: climate risks, impacts, adaptation, and the culture of citizen participation

In Malaysia average temperatures and sea-levels are rising, with an increasing variability in rainfall patterns and a growing frequency of extreme weather events (Tang, 2019). Future projections up to 2100 indicate that these trends will continue. For example, in north Malaysia annual rainfall and maximum temperature are projected to increase respectively by 1.2–8.7% and 0.6–2.1°C, implying a monthly rainfall increase during the wet season and a decrease during the dry season (Tan et al., 2017). Some north-eastern areas are highly vulnerable to sea-level rises and storm surges, due to their geomorphology, coastal slope, shoreline erosion, and tidal change (Mohd et al., 2019).

According to a literature review by Tang (2019), most existing research has focused on the potential impacts of rainfall variability and climate extremes on the agricultural sector, particularly on rice production (Tang, 2019). However, climate change is also known to influence negatively public health through mosquito-borne diseases (e.g. dengue fever) as a consequence of increased rainfall (Tang, 2019). Furthermore, Shaffril et al. (2017) highlighted the negative effects of climate change on the fishing community. Two examples are the extreme winds and waves that are expected to reduce the number of operating days, as well as associated tourism activities, and the sea-level rise that is causing coastal and mangrove erosion, as well as threatening community settlements and other infrastructure facilities.

On the adaptation policy side, academics have expressed concern that climate adaptation policies are not as well developed as mitigation policies (Khailani and Perera, 2013; Tang, 2019). For example, disasters related to climate change are not properly recognized as a risk to sustainable development (Khailani and Perera, 2013). Moreover, the vulnerability of human settlements in valleys and coastal areas is not referred to, nor is the adaptive capacity of people considered in any detail (Khailani and Perera, 2013). Certain plans - such as the Vector-Borne Disease Control Programme - may be seen as adaptation plans but which have not adopted the latest climate projection trends (Tang, 2019).

It has also been stressed that climate change has not adequately been mainstreamed into sectoral and local policies. For example, Ramakreshnan et al. (2019) point out that the “urban heat island” effect is poorly addressed in many local urban policies, while Khailani and Perera (2013) highlight vulnerability to multiple hazards has not been explicitly adopted in the districts' local plans. The latter authors also mention that disaster risk reduction should be an essential strategy to be considered at local level. Other studies indicated that there is a lack of connection between those adaptation strategies that have already been mainstreamed (Tang, 2019).

On the positive side, it has been recognized that planners have identified vulnerable zones for buildings and safe zones for emergencies (Tang, 2019). The national government is supporting research to formulate a coastal vulnerability index that could help to implement actions to tackle sea-level rise (Khailani and Perera, 2013). Similarly, there are sectoral plans tackling flood and drought risks (Tang, 2019). According to Zen et al. (2019), local climate policies are translating the national climate adaptation plan by top-down and bottom-up approaches designed to fill the gap between local and national authorities.

Regarding the culture of stakeholder and citizen engagement, it is apparent that there is a passive behaviour of the general public towards unsustainable development plans, in that they tend not to express opinions and objections (Dian and Abdullah, 2013; Ramakreshnan et al., 2019). For example, a study analysing the management of the marine zone invited 200 participants, representing a range of occupations. However, only 166 were willing to be interviewed in an initial contact. Of these, only 128 people attended a second session where a Focus Group session was organized (Abdullah et al., 2016). Other studies have adopted stakeholder engagement activities without indicating abnormal stakeholder interactions (Aziz et al., 2016; Jamil and Fathi, 2016).

Midin et al. (2017) highlight a low level of disclosure of information on Malaysian local authorities' web-sites on stakeholder engagement regarding sustainable development plans. In academic circles, however, it is recognised that good governance practice requires support from active community participation (Khailani and Perera, 2013; Zen et al., 2019). For instance, the design of adaptation measures may benefit from the engagement of indigenous communities in those forums where community members and external stakeholders come together to share perceptions and to reach consensus (Gevelt et al., 2019). As indicated by Shaffril et al. (2017), the involvement of the fishing community in climate adaptation planning can play a crucial role in the proposal of solutions.

## 3 Material and methods

The exercise developed during the training in Malaysia consisted of a Focus Group session, where the participants were asked to discuss the optimal level of stakeholder and citizen participation (following Arnstein, 1969) that should be applied to the different steps of adaptation planning proposed by the GCoM initiative (Covenant of Mayors for Climate and Energy, 2016). As explained in the following sections, this activity has been carried out by means of four Focus Groups in one session of two-and-a-half hours. In the following sections, the exercise is explained in detail, and supported by a literature review on the matter.

### 3.1 The exercise framework

#### 3.1.1 Arnstein's ladder of citizen participation

**Fig. 1:**
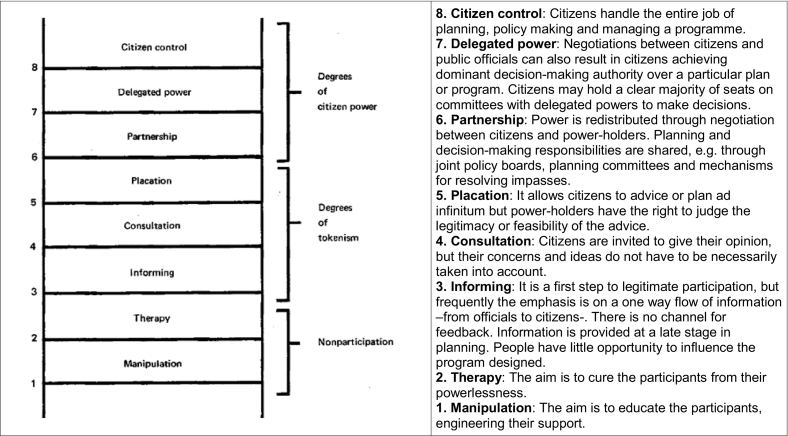
Eight rungs on a ladder of stakeholder and citizen participation. Source: Arnstein (1969).

Levels of citizen participation have been widely investigated in the literature, with numerous approaches proposed over time. Arnstein (1969) established a typology of eight levels of participation (Fig. 1), which were arranged in a ladder pattern with each rung corresponding to the extent of citizens' power in determining the final decision. Schwertz (1979) presented four levels: (1) citizens and stakeholders do not participate at all; (2) participation is only symbolic; (3) citizens influence planning as much as officials; and (4) citizens take over the process. André et al. (2006) define three levels: (1) participation is passive - citizens only receive information; (2) participation through consultation - citizens' opinions are taken into account; and (3) interactive participation - there is feedback between planners and stakeholders and citizens by means of workshops, negotiation, mediation and other integrative methods. As described in IAP2 (2014), the International Association for Public Participation launched their public participation spectrum, where citizens can be: (1) informed; (2) consulted; (3) involved; (4) invited to collaborate; or (5) empowered. Concretely regarding climate adaptation, Sarzynski (2015) proposed six levels of stakeholder and citizen participation in cities: (1) traditional government-led climate planning; (2) non-governmental planning; (3) inclusive planning; (4) partnerships; (5) non-governmental provision; and (6) co-production.

Some limitations to Arnstein's approach were highlighted by Shaw and Corner (2017), who argue that the Arnstein's typology (Fig. 1) might be of limited applicability to socialize the climate debate, especially in conservative social contexts. Hurlbert and Gupta (2015) split Arnstein's ladder into four quadrants so that the different relevance and impacts of participation could be better conceptualized and adapted to different case studies, meaning that those levels are not a one-size-fits-all. Despite its inherent limitations, and considering all the pros and cons, Arnstein's ladder of stakeholder and citizen participation was used in the exercise carried out in Malaysia, since it provides detailed levels of citizen participation.

#### 3.1.2 Steps of adaptation planning in the Global Covenant of Mayors

The Covenant of Mayors (CoM) “2020 target” initiative was launched in 2008 by the European Commission to support the efforts deployed by local authorities in the implementation of sustainable energy policies. The local authorities' commitment was to achieve by 2020, at least the European 20% reduction of the total emissions objective compared to the baseline, in the area of influence of the local authority, by the implementation of a Sustainable Energy Action Plan (SEAP). In parallel, in 2014, in the context of the European Strategy on adaptation to Climate Change,^3^ the European Commission launched a separate initiative called “Mayors Adapt”, based on the same principles as the CoM, which focused on adaptation to climate change and on the support to local authorities in the development and implementation of local adaptation strategies (Bertoldi, 2018). In 2015, the CoM and Mayors Adapt initiatives merged into the Covenant of Mayors for Climate and Energy that adopted a threefold vision: reducing greenhouse gas emissions; strengthening the capacity to adapt to unavoidable climate change impacts; and ensuring universal access to secure, sustainable and affordable energy services for all.

In 2017 the Global Covenant of Mayors for Climate and Energy initiative was launched, bringing under a harmonised approach the commitments of local governments originally presented either through the Compact of Mayors^4^ and the pre-existing Regional / National Covenants of Mayors and newly developing Regional / National Covenants. A set of new common recommendations (Common Reporting Framework - CRF) allows signatories to operate under the shared vision of the GCoM with principles and methods that best suit their region (Bertoldi, 2018). The CRF covers the key elements of GCoM (emissions inventories, targets, climate risk assessments and climate action plans) and allows consistency with national and/or sub-national requirements for local governments within their own context. The implementation of the GCoM in Asia is supported through the International Urban Cooperation (IUC) Asia project. Six Malaysian local authorities have already joined the GCoM.^5^

**Fig. 2:**
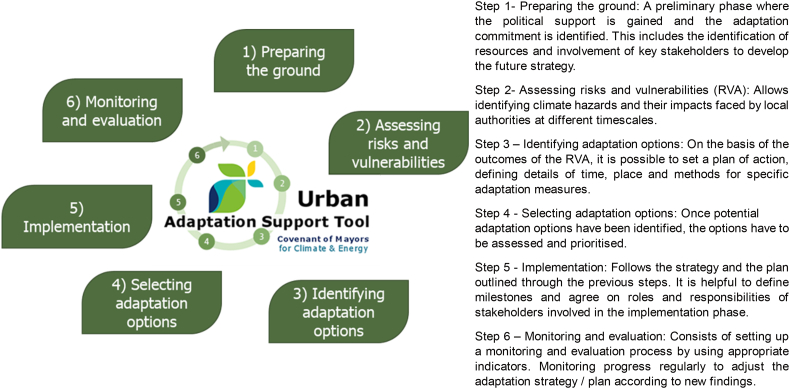
The climate adaptation planning cycle. Source: https://climate-adapt.eea.europa.eu/knowledge/tools/urban-ast/

Within the adaptation pillar, the Urban Adaptation Support Tool (UAST) (see Fig. 2), was developed by the European Environmental Agency and the European Covenant of Mayors to help signatories to enhance their climate resilience. Adaptation planning was consequently organized in a cycle, as shown in Fig. 2, where the key steps for developing an adaptation strategy are identified. This climate adaptation planning cycle was the one proposed to the participants of the training as part of the exercise described in this paper.

#### 3.1.3 Focus groups technique

Focus Groups are a form of group-interviewing aimed at collecting qualitative information to answer research questions (Bloor et al., 2001). Focus Groups are helpful tools to learn more about a certain subject, and to discover actors' opinions with regards to the research questions (Morgan and Krueger, 1993). Focus Groups also assist researchers to identify attitudes, feelings, beliefs, experiences, and reactions that would not be possible to detect through other social research methods, such as observation, one-to-one interviews or questionnaire surveys (Gibbs, 1997).

Focus Groups are not exempt from bias and drawbacks. For example, with Focus Groups it can be difficult to obtain representative samples (Gibbs, 1997). Therefore, Focus Groups might not provide accurate information on what a whole community thinks about a certain topic (Gamboa and Munda, 2007). The moderator / researcher may also lose control of the interaction and it may be complicated to identify an individual message (Gibbs, 1997). Focus Groups may discourage some participants since the method does not provide confidentiality (Gibbs, 1997), or because some topics may be unacceptable for discussion (Morgan, 1996), or due to differences in social status that may cause silence among some participants (Bloor et al., 2001). The opposite may also occur, in that certain participants might monopolize the discussion (Smithson, 2000), perhaps due to power relations (Bloor et al., 2001). Hernández González (2014) has also indicated that when the participants are too polarised, Focus Groups might not work adequately.

Although the authors of the research described in this paper had control over the interaction and avoided monopolization from some participants during the facilitation process, it cannot be guaranteed that participants were, to a certain point, discouraged due to lack of confidentiality, differences in social status or due to established power relations. However, none of these drawbacks were perceived.

The climate adaptation research community has applied this social technique for diverse purposes. Lennox et al. (2011) used it to evaluate criteria assessment for water resource management. Nagoda and Nightingale (2017) used it to demonstrate how social and power relations perpetuate the vulnerability of certain social groups to climate change. Other studies have examined the role of gender in the perception of climate risks and the proposal of adaptation strategies (Ngigi et al., 2017), as well as the understanding of the concept ‘resilience’ in relation to tree pests and health (White et al., 2018).

In the exercise described in this paper, four Focus Groups were organized to discuss the level of participation desired by civil servants according to their needs and the specificities of the local authority they worked for.

### 3.2 The exercise in Johor Bahru

#### 3.2.1 The training session

**Fig. 3:**
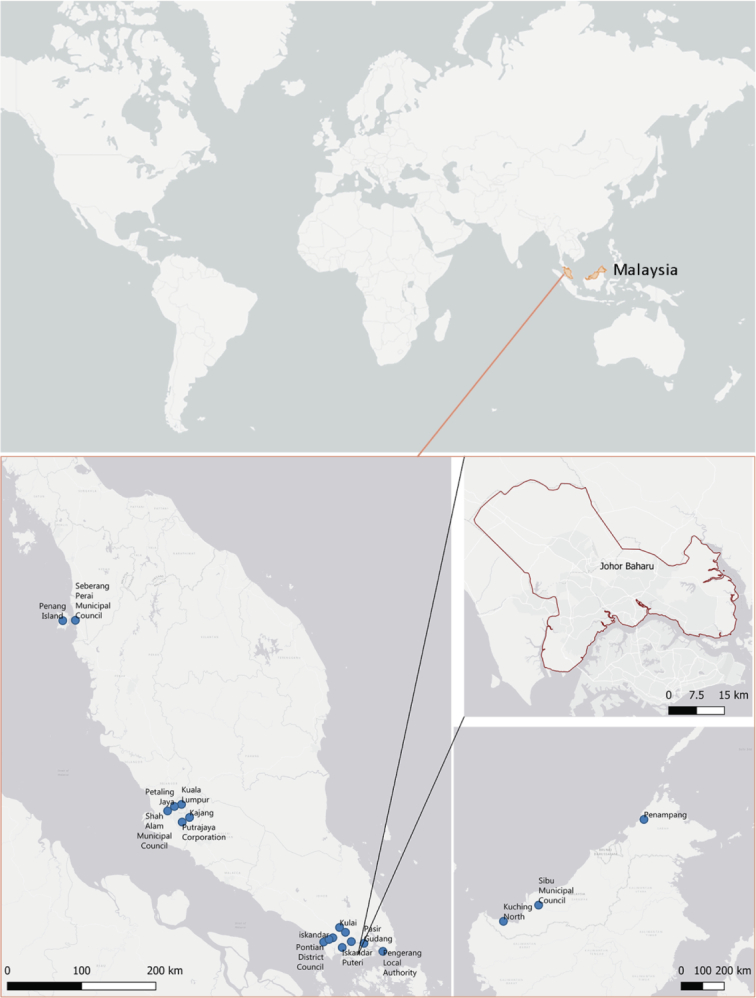
Maps showing the cities participating and Johor Bahru District, Malaysia. Map source: JRC (elaboration by author).

In March 2019, a training on climate adaptation was held in the city of Johor Bahru in Malaysia, in the Iskandar region.^6^ Johor Bahru (Fig. 3) is a municipality of around 1 million inhabitants, which is becoming a more production- and services-based economy due to the massive development activities of the Iskandar Malaysia project city plan (Abba et al., 2013).

The training session was part of the planned GCoM-related activities of the International Urban Cooperation (IUC) Asia programme. IUC Asia, in collaboration with GCoM and the Iskandar Regional Development Authority (IRDA), organized a three-day GCoM Workshop on climate adaptation dedicated to civil servants from Malaysian municipalities and regional departments. Affiliations of the participants included: IRDA; Sibu; Shah Alam; Seberang Perai; Putrajaya; Pontian; Petaling Jaya; Pengerang; Penang; Penampang; Pasir Gudang; Kulai; Kuching North; Kuala Lumpur; Kajang; Johor Bahru; Iskandar Puteri City Council; PLAN Malaysia.

Numerous experts from universities, non-governmental organizations (NGOs) and international organizations participated in this event, as key interested stakeholders. A thematic discussion and networking event followed the training to share information on the GCoM initiative and promote local climate action in the region.

The three-day Workshop was organized in several sessions that covered all the key steps of the adaptation climate adaptation planning cycle (Fig. 2), with interactive activities allowing participants to focus on the topics in a proactive manner. All participants were fluent in English, so there was no need for translation, which made the discussions more direct and active. On the first day, after introducing the key concepts of adaptation planning, one activity was the Focus Group exercise that was led by the European Commission's Joint Research Centre (JRC) and upon which this study is based, and which is described in detail below. More information on the training is available at: https://www.iuc-asia.eu/2019/03/training-on-adaptation-in-johor-bahru-malaysia/.

#### 3.2.2 The four focus groups and the activity

The Malaysian local governments represented at the training were from different levels of the climate adaptation planning cycle (Fig. 2). More advanced municipalities had already developed an adaptation plan or risk assessment, while others were approaching the first steps of the adaptation cycle. During the whole event there were about 60 participants, of whom around 40 took part in the Focus Group session. The Focus Groups were facilitated by the authors of this paper, who invited IUC Asia to form four groups of about 10 persons each with the aim of maximising heterogeneity in terms of working municipality, gender, age, and background.

All of the participants were first given a hand-out illustrating Arnstein's ladder and their definitions (Fig. 1) and a copy of the climate adaptation planning cycle (Fig. 2), in order for them to link the levels of stakeholder and citizen participation with each step of the cycle. Participants were invited to read the documents carefully before the session began and to request clarification if needed.

A 15 min period of the session was used by the authors to introduce and explain the dynamic of the exercise. Participants had a further 15 min to read the hand-outs and to request clarification. Next, the four Focus Groups deliberated for an hour to answer the following four main questions:

1. *Who are the stakeholders and citizen organizations that should be involved in adaptation planning in the region?*
2. *What level of citizen participation would they assign to each step of the adaptation planning?*
3. *What social techniques/methods would they implement to reach those participation levels?*
4. *Which barriers have they encountered or expect to find in the engagement process?*

Finally, each group had 15 min to present its results and to answer questions from the other groups. The Focus Groups decided to lead the exercise, taking inspiration from a concrete situation of risk that could occur in the local territory. All the information produced and shared was recorded (with participants' consent), photos were taken and proposals were collected as raw material.

## 4 Results

### 4.1 Stakeholders and citizens to be involved in the adaptation planning (Results for Question 1)

**Fig. 4:**
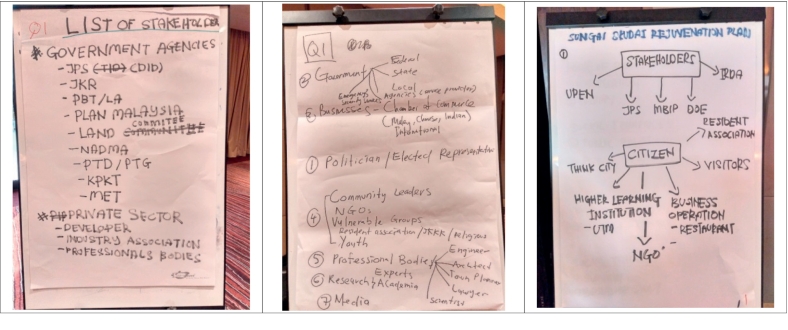
Examples of the flip-chart notes recorded during the discussions on Question 1 by Focus Groups 1 (left), 2 (centre), and 4 (right). Note: Focus Group 3 did not use a flip-chart.

In order to answer the first of the four questions listed in Section 3.2.2, the Focus Groups provided a list of potential stakeholders and/or citizen organizations, with an explanation of their choice. Government agencies at national and local levels, industrial associations and professional bodies, academy and NGOs were the most frequent selected potential stakeholders. The specific results from each Focus Group in relation to Question 1 are listed in Table 1, and examples of flip-chart notes made by the Focus Groups during their deliberations are shown in Fig. 4.

**Table 1:** List of stakeholders and citizen organizations that should be involved in climate adaptation planning, as proposed by the four Focus Groups in response to Question 1 (see Section 3.2.2).

Stakeholder category	Focus group 1 (composed of 9 persons:6 men, 3 women)	Focus group 2 (composed of 10 persons: 8 men, 2 women)	Focus group 3 (composed of 9 persons: 2 men, 7 women)	Focus group 4 (composed of 7 persons: 3 men, 4 women)
Public sector:	**Politicians & decision-makers.**	**Politicians & decision makers:**	**Agencies:**	**Agencies:**
			* Government agencies (local authorities & implementation contractors).	* Government agencies (UPEN/State economic affairs unit; JPS/Department of irrigation and drainage).
		* Community leaders.		
	**Agencies:**	**Agencies:**		
	* Government agencies.			
	* Local agencies.	* Federal agencies.		
		* State agencies.		
		* Local agencies.		**Departments:**
	* National disaster management agency.	* Emergency & security agencies.		* Department of private education.
	* Local government - meteorological agency.			
				* MBIP (Iskandar Puteri City Council).
	**Departments:**			* IRDA (Iskandar Regional development authority.
	* Housing department.			
	* Disaster risk management department.			
				* Department of environment.
	* Public works department.			
	* Disaster management committee.			
	* Common public committee.			
Private sector:	**Associations:**	**Associations:**	**Associations**:	**Companies:**
	* Industries association.	* Chambers of commerce (Malay, Chinese, Indian, international).	* Private agencies.	* Business operation.
	* Professional bodies.		* Business committees.	
			**Companies**:	
		* Professional bodies (engineers, architects, town planners, lawyers).	* Developers.	
			* Corporations.	
NGOS & research institutions:	* Academics.	* Vulnerable groups.	* Residents associations & activists.	* Residents associations.
		* Residents associations.		
				* Academics (UTM/University of Technology, Malaysia).
		* Researchers, experts & academics.		
Media:	–	* Media.	* Media.	–
General public:	–	* Individuals (youth).	* Individuals (youth and women).	* Citizens.
				* Visitors/tourists.

Focus Group 1 put a particular emphasis on the public sector compared with the other groups: “For our group, … the first one is the Government Agencies …: Inundation Department and Public Works Department, Local Agencies, National Disaster Management Agency, Housing and Local Government Agency, Meteorology Asia, and a few others such as the Disaster Management Committee”. Nine public sector organizations were identified, compared with an average of two for the other three groups.

Focus Group 2 highlighted the need to involve young people as a way to shape the vision of the future: “We don't know what they want”. They also subdivided the business sector into four groups representing the main ethnic groups in the country: Malaysian, Chinese, Indian and international. Focus Group 2, as well as group 3, selected the media as a key stakeholder, identifying its potential for dissemination and communication.

Focus Group 3 identified individuals as stakeholders, organized in groups with different functions (e.g. youth and women) in order to highlight diverse perspectives.

Focus Group 4, unlike the other groups, listed tourists and visitors among the above-mentioned stakeholders.

### 4.2 The level of citizen participation per each step of the adaptation planning and related social techniques (Results for Questions 2 and 3)

**Fig. 5:**
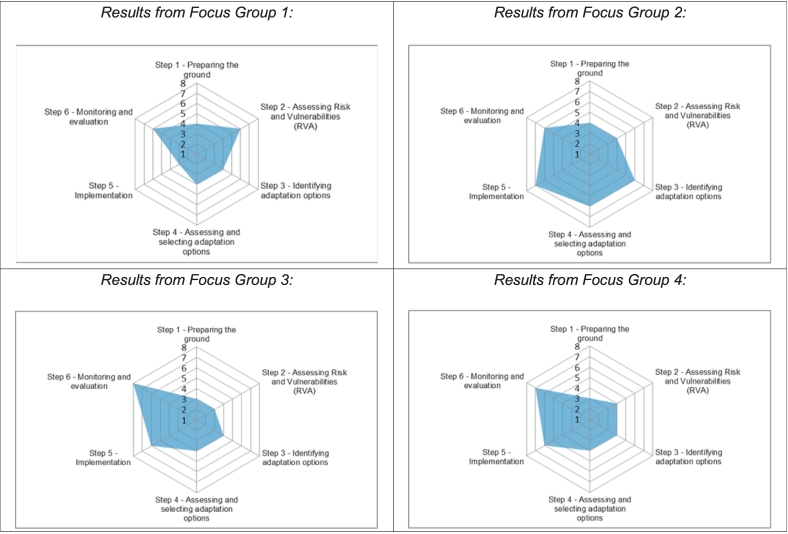
“Spider plot” graphs showing the different levels of citizen participation assigned to each step of the climate adaptation planning cycle, as proposed by each Focus Group in response to Question 2 (see Section 3.2.2). The numbers 1–8 refer to the levels of engagement defined by Arnstein (see Fig. 1).

Questions 2 and 3 (see Section 3.2.2) were addressed together by each of the Focus Groups, since the process of considering the level of stakeholders' involvement for the different steps of the climate adaptation planning cycle was more straightforward if combined with a reflection on the methods used to reach the selected level. Fig. 5 shows the results regarding Question 2 presented as spider plots, where each graph shows the answers agreed by each group after the discussions.

For Step 1 *(Preparing the ground)*, all Focus Groups selected medium / low levels of citizen participation, i.e. from *Informing* to *Consultation.* This was interpreted as a way to “appoint” people who know the area at risk. For example, Focus Group 1 stated that “through consultation, we should appoint consultancy that can really know the area that will be impacted by flooding. … That is professionals' opinions come together with the participation of the local people”. In this case, the common technique was the survey, supported by social media.

For Step 2 *(Assessing risks and vulnerabilities)*, a higher involvement of stakeholders was considered appropriate, in particular for Focus Group 1 that selected *Partnership*: “should be done, really should be done, through Partnership, because it is not only the consultation of professionals that should look into it, the common people know better their area. […] They know the vulnerable areas”. In this case, the Focus Group rapporteur highlighted the fact that the selection of that level was driven by consideration of integrating the technical approach of professionals with that of citizens who might have a non-scientific knowledge of the territory and the issues at stake. In fact, *Consultation* was mainly perceived as a method to engage experts and scientists who can be asked for technical matters. All the other Focus Groups selected the *Consultation/Informing* level. Regarding social techniques, Focus Group 1 suggested running Focus Groups and proposed “Safety Walks” “for bringing together all the community people to the streets, start walking and identifying points [of risk]”. Safety Walks might be considered a bottom-up approach that brings together different stakeholders with a common objective (discussed in Section 5.3 below). The other Focus Groups agreed on surveys and baseline studies.

Steps 3 *(Identifying adaptation options)* and 4 (*Selecting adaptation options*) were treated simultaneously and were assigned the same level of engagement. In particular: Focus Groups 1, 3 and 4 selected the *Consultation* level, since the potential actions included in these steps were perceived as very technical and, hence, requiring specific expertise. Workshops and Open Days were selected as the most suitable social techniques. Focus Group 1 suggested the so-called “*dotmocracy*” approach (also known as dot democracy, dot-voting or voting with dots), which is an established facilitation method that allows expressing votes with dot stickers or marks with a marker pen. For these steps Focus Group 2 assigned the level of *Partnership* for actively engaging local communities.

Steps 5 (*Implementation*) and 6 (*Monitoring*) were attributed the highest level of citizen participation. For Step 5, Focus Group 2 assigned the level of *Delegated power*, associated with dialogues, Focus Groups and facilitating processes. In contrast, for Step 5 Focus Group 1 assigned to the level of *Informing*, thus leaving higher involvement for the decisional rather than the implementation stage: “They [decision-makers] have to decide in power whether they want this project or not. So when they decide to do that particular project, they should inform the people”. This is proposed to be put in place through informative sessions, including a Kick-Off Meeting.

Focus Group 3 considered *Consultation* and *Partnership* as the most suitable levels of engagement for implementing the adaptation action plans, since governments need support and cooperation to be effective. Similarly, Focus Group 4 referred to *Partnership* for Step 5, in particular focusing on financing and funding adaptation actions. Outreach promotion and awards were suggested as social techniques.

Step 6 is mainly assigned to stakeholders who, having followed the whole process with different levels of engagement, can have a clear understanding. Focus Group 1 planned to monitor the status of the adaptation strategies through the *Partnership* level, in particular with joint committees, described as biannual meetings of Heads of Department in charge of climate adaptation affairs, and dialogue approaches. Focus Group 2 also identified *Partnership* as suitable: “We cannot believe and rely on governments for this climate action plan. So we would like to have more involvement like NGOs and businesses, because we are from governmental agencies and we know our limitations”. Focus Groups 3 and 4 assigned higher levels - *Citizen control* and *Delegated power*, respectively - to be implemented through impact assessments.

#### 4.2.1 Steps 1–6 – Social techniques/methods to reach participation levels

A summary of the results arising from the deliberations of each Focus Group regarding both Questions 2 and 3 (see Section 3.2.2), is provided in Table 2. As can be seen, the most common levels of stakeholder and citizen engagement in numerous stages of the adaptation planning cycle are *Informing*, *Consultation* and *Partnership*. Similarly, according to the participants, Focus Groups are among the most amenable social techniques for involving citizens and stakeholders.

**Table 2:** Summary of the various social techniques and methods to implement the desired citizen participation level, as proposed by the four Focus Groups in response to Question 3 (see Section 3.2.2).

Steps in planning cycle	Focus group 1	Focus group 2	Focus group 3	Focus group 4
1. Preparing the ground	Consultation with professionals and local people using surveys.	Consultation through social media.	Informing.	Informing through public engagement.
2. Assessing risks and vulnerabilities	Partnership through Focus Group discussion and Safety Walks.	Consultation with professionals.	Informing.	Consultation through surveys and baseline studies.
3. Identifying adaptation options	Consultation with professionals and local people through technical committees.	Partnership through Focus Groups.	Consultation.	Consultation through Focus Groups and Workshops.
4. Selecting adaptation options	Consultation through dotmocracy (see text).	Partnership.	Consultation.	Consultation through Open Days.
5. Implementation	Information through Kick-Off Meetings.	Delegated power through dialogues and Focus Groups.	Partnership.	Partnership.
6. Monitoring and evaluation	Partnership through joint committee meetings.	Partnership through check and balance.	Citizen control.	Delegated power through impact assessments.

### 4.3 Barriers encountered or expected in the engagement process (Results for Question 4)

The responses of the Focus Groups to Question 4 (see Section 3.2.2), which focused on the barriers already encountered or expected when developing the adaptation strategy, are summarised and harmonised in Table 3, and briefly discussed below:

**Table 3:** List of the barriers encountered or expected in the engagement process, as identified by the four Focus Groups in response to Question 4 (see Section 3.2.2).

Focus group 1	Focus group 2	Focus group 3	Focus group 4
* How to manage expectation/frustration.	* Limited resources and financial constraint.	* Resistance to adaptation.	* Coordination and integration among stakeholders.
* Time management.	* Communication gap.	* Coordination and integration among stakeholders.	
			* Knowledge gap.
			* Behaviour and attitude.
* Communication gap.	* Professional arrogance.		
* Hidden political agendas.			
	* Skepticism.		
	* Community perception.		
	* Social empathy.		
	* Personal interest.		

As shown in Table 3, communication and knowledge gaps are perceived as a considerable limit. For example, Focus Group 1 stated: “We, as officials, tend to have some kind of ideas, and kind of thinking. It might not be the same for the communities. … We might see something that they don't understand and they might see something that we can't understand”. This might also be considered as difficulties to share opinions and points of view and as a matter of difficulty of language.

Related to communication gaps, Focus Group 2 stated that the community perception of the activities developed by local governments might differ and hamper effective adaptation: “We cannot use all this [technical] language, because community will not understand”. Behaviour changes and people expectations were also highlighted as challenges.

Focus Groups 3 and 4 highlighted stakeholders' engagement and methods to coordinate and develop outcome from this process. For example, according to Focus Group 3: “Our barriers are resistance to adapt - … it is hard for us to bring the citizens to changes”. Finally, time and financial constraints were highlighted as barriers to the practical aspects of plan development and implementation.

## 5 Discussion

In this section, the main patterns detected in the results presented previously, are discussed. Up to four patterns have been identified, as well as one possibly new social technique - the “Safety Walks” - that may merit further attention. The first pattern discussed concerns the stakeholder and citizens organizations to be engaged in climate adaptation planning. The second pattern relates to the levels of public engagement for all steps in the adaptation planning cycle. The third pattern refers to the social techniques to be applied for specific levels of engagement. Finally, the fourth pattern pertains to the barriers encountered in carrying out the participatory processes.

### 5.1 Stakeholder and citizen engagement in climate adaptation planning

As was highlighted in Section 4, all Focus Groups agreed on the need to engage, at the very least, the following stakeholder and citizen organizations: government agencies; business organizations; NGOs; and neighbourhood associations. Government agencies at all levels of governance (national, regional, and local level) are frequently mentioned as a key actor in the existing literature on stakeholder engagement for adaptation (Melica et al., 2018). Sandink et al. (2016) indicate that municipal staff, conservation authorities, provincial government, federal government, and decision-makers are those involved in the development and design of decision-support systems for updating and incorporating climate change impacts. Similarly, a case study carried out in Kuala Lumpur (Malaysia) engaged policy-makers and practitioners of urban planning in Urban Heat Island adaptation (Ramakreshnan et al., 2019).

Moreover, although the groups may differ in how it is named, there is consensus on the fact that the business sector should also be involved in adaptation planning. This stakeholder group includes consulting firms, utility organizations, financial industry or industry associations (Sandink et al., 2016). NGOs are the other type usually mentioned in the literature on stakeholder engagement in adaptation planning – in particular NGOs representing the most vulnerable social groups. The need to engage NGOs has been documented by Sandink et al. (2016) and Ramakreshnan et al. (2019). Lastly, three of the Focus Groups indicated that neighbourhood associations, especially those largely exposed to climate hazards, should be taken on board in climate adaptation planning, as is underlined also in the existing literature. For instance, Marfai et al. (2015) engaged the neighbourhood association in Jakarta (Indonesia) in the search of community responses and adaptation strategies to mitigate flood risks.

The results, therefore, reinforce what the existing literature points out with regards to the need to engage key stakeholder and citizen organizations (see also the last paragraph of Section 2). In this regard, not much added value is provided to the scientific community.

### 5.2 Levels of public engagement for all steps in the climate adaptation cycle

The results regarding levels of citizen participation, indicate a possible agreement by all Focus Groups on a minimum engagement level - such as *Informing* and *Consultation* - that could be implemented during the first four steps of the climate adaptation planning cycle (see Fig. 2). However, Focus Groups 1 and 2 suggested that *Partnership* could also be explored for assessment of climate risks and identification of adaptation options (i.e. Steps 2 and 3).

In general, the existing literature on stakeholder and citizen participation dissents from these results, since *Consultation* is actually considered insufficient for developing good adaptation planning. Mielke et al. (2016) consider *Consultation* engagement as a technocratic type of participation where stakeholders and citizens are only seen as a source of information, implying a one-direction flow of information and knowledge. Shaffril et al. (2017) analysed diverse adaptation strategies for small-scale fishermen in Malaysia, adopting higher levels of stakeholder and citizen participation than *Consultation*. Similarly, Masud et al. (2018) state that the Malaysian government should intensify the level of stakeholder and citizen engagement in flood risk management plans. However, it is also true that Focus Group 1 considered that *Partnership* should be adopted for assessing climate risks (i.e. Step 2), whereas Focus Group 2 declared *Partnership* for identifying and selecting adaptation options (i.e. Steps 3 and 4).

However all Focus Groups were also able to agree on a *Partnership* level of participation for implementation (Step 5) as well as *Partnership / Delegated power* for monitoring and evaluation (Step 6). These two higher levels of participation are considered a democratic type of participation (Mielke et al., 2016), since the integration of all stakeholders and citizens interested in the process is rooted in a collaborative approach where the problem definition is co-designed, solutions are co-produced and implementation is co-validated.

Thus, according to the Focus Groups, the more advanced the climate adaptation plan is, the higher the level of stakeholder and citizen participation that should be adopted. The reason behind this shall be linked to the need for integration to overcome limitation. As indicated in section 4.2, Focus Group 2 explained that “we cannot believe and rely on governments for this climate action plan. So we would like to have more involvement like NGOs and businesses, because we are from governmental agencies and we know our limitations”. Focus Group 3 also declared a similar reason: “we government cannot do everything alone, so we need to partner with all stakeholders”. Lastly, Focus Group 1 pointed out that the adaptation plan “should be monitored by everyone … The monitoring should be done by the Government Agency, … the private sector and … the people through joint committee meetings”. These statements are consistent with so-called social sensitivity analysis theory (Corral and Acosta, 2017; Corral and Hernandez, 2017), according to which the results obtained in a participatory process have to be given back to the community. Through this process, stakeholders have the chance to propose corrections and make suggestions to improve the developed assessment, thereby running a quality check. Transparency and shared knowledge produced through higher participation levels improve the robustness of the whole process.

Stakeholder and citizens should be involved in the whole process, with a tailored approach according to the local specificities, as also highlighted by Wamsler (2017) and Sarzynski (2015). In fact, the Focus Groups tended to put more emphasis on the latest steps of the adaptation planning cycle (i.e. implementation and monitoring). This high level of stakeholder and citizens engagement in the latest steps of the planning cycle might be a novel result for the climate adaptation community that opens for new lines of reflection and research. In the case of Malaysia, this result would be useful to improve the passive behaviour of the general public regarding participation in sustainable planning (see Section 2).

Furthermore, it is believed that this result may relate to avoiding conflict (Kovács et al., 2016), presenting additional benefits for the adaptation policy cycle. It is well known that an ample information provision in the initial steps of the policy cycle is of great importance to ascertain major conflicting values and to find compromise solutions (Kamlage et al., 2020; Siebenhüner, 2018). However, conflicts in adaptation policy can emerged in the latest steps if stakeholders feel they did not contribute with their knowledge to the final products of the project (Siebenhüner, 2018). Thus, it could therefore be inferred that a participatory process with an increasing level of involvement could contain the emergence of conflicts.

### 5.3 Social techniques and methods to reach engagement levels

While some agreement on the level of engagement was identified, there was no clear consensus among the Focus Groups on which social techniques and methods should be adopted to reach the desired participation level. Focus Group discussions were mentioned as a desirable social technique for *Consultation*, *Partnership* and *Delegated power*. In addition, surveys were mentioned as a preferred social technique for *Informing* and *Consultation.* Despite being a vague concept, dialogues were also considered a useful social technique for *Informing*, *Partnership* and *Delegated power*. Lastly, Joint Committees (see Kittikhoun and Staubli, 2018) were suggested to be applied to *Consultation* and *Partnership.*

Therefore, while, according to the Focus Groups there is no consensus or pattern regarding the techniques for the two levels *Informing* and *Delegated power*, *Consultation* could be developed using surveys and expert groups. Meanwhile, *Partnership* may be adopted through Focus Group discussions.

Finally, the “Safety Walks” method was mentioned by Focus Group 1 for carrying out the assessment of climate risks. Safety Walks are a risk management technique designed to deal with public hazards, by means of a standard checklist to enhance the safety of pedestrian areas and businesses (Gnoni et al., 2013; Powell et al., 2000; Webb, 2009). Safety Walks are a well-known method to guarantee safety in many social and economic sectors, for example: in air transport, to check aircrafts before take-off (High et al., 2005); in the health sector, to monitor patient wellbeing (Ayuso-Murillo et al., 2017; Behm et al., 2014; Shaw et al., 2006); on construction sites, to minimize on-site risks (Chan et al., 2010; Choi et al., 2011; Choi et al., 2012; Dennerlein et al., 2009; Langfield-Smith, 2008; McDonald et al., 2009; Oswald et al., 2018; Oswald and Lingard, 2019; Rowlinson and Jia, 2015; Winge et al., 2019; Zou et al., 2017); in port operations, as preventive maintenance activities (Gerbec and Kontic, 2017); in industrial activities (Tappura et al., 2017); in the farming sector, to control possible risks on the farm (Holte and Follo, 2018); at schools, to enhance proactive safety behaviour (Kurki et al., 2019); as well as other business activities (Zwetsloot et al., 2017).

Walks have also been used to build seismic vulnerability maps, referring to this tool as “transect walk”. Being differentiated in labels (transect walks, safety walks) and specific aims, this technique is spreading in the climate context as well and it pursues the strengthening of the role of local knowledge and the increasing confidence of locals (Charan et al., 2018; Ahmed, 2018, Naess, 2008). To this aim, this tool may complement other existing community-based tools. However, the walks approach is not free from criticisms. Berman et al. (2018, pp. 383) reported the skepticism of an interviewee regarding the impact of Safety Walks: “Administrators value ‘safety theatre’ - the appearance of caring about safety. They don't seem to know what it actually is beyond slogans and cute acronyms. None of these activities has resulted in tangible improvements. Safety walk-rounds are the worst of examples of ‘safety theatre’. Ideology replaces observation. Of the tools that are available, none has led to new systems that demonstrably decrease error or improve safety”). Other cases showed how potential bias araises from safety issues, selection of participants and the specific framework in terms of time and place (Kanstrup et al., 2014). Despite the differences in sectors and procedures, walking through the community to identify issues and suggest methods to address them gives voice to locals and increase the knowledge of researchers. Community-based climate change adaptation, based on the existing literature on the topic, is gaining momentum. Thus, “Safety Walks” could be considered as a currently spreading and innovative participatory technique whose potential successful outcomes need to be further investigated and its implementation in climate risk assessments explored (i.e. Step 2 in Fig. 2).

### 5.4 Barriers encountered or expected in carrying out the participatory processes

The results from the Focus Groups indicate some apparent patterns with regards to the possible barriers identified when implementing participatory processes in adaptation planning. According to the Focus Groups, two main categories of barriers may be discerned: (a) communication and knowledge gaps; and (b) problems with how to integrate and coordinate stakeholders and citizens and keep them all participating throughout the process. These barriers have already been identified by other practitioners of environmental planning (see below), which highlights how common issues are perceived and faced also in the framework of the climate adaptation, implying a certain novelty for this field of knowledge.

Regarding communication and knowledge gaps, Murombo (2008) highlighted the fact that public participation can be hampered due to the issue of language, since in most cases project documents use technical and complex language that may not be understood by all interested stakeholders and citizens. Similarly, Lindenau and Böhler-Baedeker (2016) stated that inappropriate tools, such as an unadapted type of involvement for each stakeholder group, may lead to an ineffective participation. Rivas et al. (2016), in analysing methods to reduce energy consumption, pointed out the high potential of information campaigns in bridging the communication gap. These could be shaped in ways that are able to target different groups and cover several sectors.

The challenge of how to integrate and coordinate the participatory process has also been mentioned by the literature. Loring (2007) pointed out poor advertisement, inconvenient location, intimidating locations for some individuals, and difficult time schedules. Meanwhile, Lindenau and Böhler-Baedeker (2016) suggest the lack of skills when preparing and leading a participatory process by the organizers and facilitators.

Lindenau and Böhler-Baedeker (2016) also identified other possible drawbacks, such as: (1) lack of political support to commit to in-depth participation; (2) limited institutional resources to set-up a successful participatory process; (3) inadequate financial resources - since there is no specific budget reserved for stakeholder and citizen participation; (4) an unclear and unrealistic strategy; (5) an incomplete and imbalanced list of stakeholders; (6) an inappropriate level of participation; (7) uncertainty about the integration of the results; and (8) underestimated administrative efforts, such as time required, data collection and analysis of inputs.

Some of the drawbacks identified by Lindenau and Böhler-Baedeker (2016) can be linked with the above-mentioned two main barriers highlighted by the Focus Groups. Moreover, these mainly relate to the lack of a systematic approach to stakeholder engagement, the establishment of which is one of the goals of the exercise described in this study.

As reported in Table 3, other drivers can influence the adaptation process, such as personal interests, community perception and attitude. The importance of personal values and world-views is known to shape engagement in adaptation (Brink and Wamsler, 2019). However, other remarkable barriers for a successful implementation of adaptation actions were not mentioned during the discussions, such as the lack of clear authority at the national scale, as pointed out by Nightingale (2017).

## 6 Conclusions

The experimental exercise that was carried out in Johor Bahru, Malaysia, during a training session to civil servants, used Focus Groups to trigger a constructive dialogue on, stimulate interest in and raise awareness of the matter of citizen and stakeholder engagement in climate adaptation planning. The activity was framed within the Global Covenant of Mayors on Climate and Energy initiative, and pursued the link between the climate adaptation planning cycle developed as part of the Urban Adaptation Tool, and the study of Arnstein (1969) on the levels of engagement of stakeholders.

Against the authors' hypothesis, the civil servants involved in the exercise tended to suggest medium to high levels of stakeholder and citizen participation in the adaptation planning process. This reinforces the assumption that when practitioners are induced to collaborate and to reason on challenging issues, results are positive and awareness is raised. The Focus Groups identified numerous stakeholders and specified their different roles in the adaptation planning cycle. They all highlighted the need for technical expertise to be integrated with the view-point of people who are living the territory and experiencing disasters. In particular, with reference to the six steps of the adaptation planning cycle (see Fig. 2), the role of individuals and citizens is required to be more active in implementation and monitoring (i.e. Steps 5 and 6). The increasing level of engagement may relate to avoiding conflict, presenting additional benefits for the adaptation policy cycle. An ample information provision in the initial steps of the policy cycle is known to be of great importance to ascertain major conflicting values and to find compromise solutions. Furthermore, conflicts in adaptation policy can also be avoided in the latest steps if stakeholders feel they contributed with their knowledge to the final products of the project. Therefore, it could be inferred that a participatory process with an increasing level of involvement might contain the emergence of conflicts.

Consequently, while the assessment of risk is mainly perceived to be a technical and scientific matter, the implementation and monitoring steps are linked with the active participation of citizens who are the so-called “users” of the city. However, the willingness to combine and integrate multiple profiles in all of the stages emerged as a key point in the discussion phase of the Focus Groups activity. In this regard, the “Safety Walks”- a social technique suggested by one of the Focus Groups – with its bottom-up integrated and participatory approach could be further explored and developed to understand its potentials and outcomes within the climate adaptation planning framework, but also applied to numerous cases linked to the territory. The implementation of the Safety Walks technique might induce the awareness-raising of citizens, stimulate their active participation, and complement climate risk assessment (which has so far been considered as a more technical step within the adaptation planning process). Furthermore, the Safety Walks could also have a positive effect in addressing several of the identified barriers in the adaptation planning participatory process, in particular: (1) communication and knowledge gaps; (2) problems with how to integrate and coordinate stakeholders and citizens and keep them all participating throughout the process; and to some extent, (3) behaviour and attitude change. The authors encourage the climate adaptation practitioners to apply and verify the potential of Safety Walks as a complement to climate models, when carrying out climate risk assessment. Its strengths and drawbacks merit further exploration.

Finally, the Focus Groups activity that was developed resulted in stimulating participants by raising site-specific and local challenging issues to which the participants themselves found answers and solutions, through an active discussion and a dynamic sharing of practices and suggestions. How and to what extent the regional features influenced the outcomes of this training session has to be verified by applying the same structure of exercise to other global regions.

## Disclaimer

The views expressed are purely those of the authors and may not in any circumstances be regarded as stating an official position of the European Commission.

## Declaration of Competing Interest

None.

## Footnotes

2 https://unfccc.int/process-and-meetings/the-paris-agreement/the-paris-agreement

3 An EU Strategy on adaptation to climate change (COM/2013/216 final).

4 Launched by Ban Ki-moon and Mike Bloomberg in 2014, the Compact of Mayors was a global coalition of city leaders addressing Climate Change by pledging to cut greenhouse gas emissions and prepare for the future impacts of Climate Change (Barron-Lopez, 2014).

5 https://www.globalcovenantofmayors.org/our-cities/

6 https://www.iuc-asia.eu/2019/03/training-on-adaptation-in-johor-bahru-malaysia/
